# Implementation of Antenatal Lifestyle Interventions Into Routine Care: Secondary Analysis of a Systematic Review

**DOI:** 10.1001/jamanetworkopen.2022.34870

**Published:** 2022-10-05

**Authors:** Mahnaz Bahri Khomami, Helena J. Teede, Joanne Enticott, Sharleen O’Reilly, Cate Bailey, Cheryce L. Harrison

**Affiliations:** 1 Monash Centre for Health Research and Implementation, School of Public Health and Preventive Medicine, Monash University, Melbourne, Australia; 2 Diabetes and Endocrine Unit, Monash Health, Melbourne, Australia; 3 UCD Institute of Food and Health, University College Dublin, Belfield, Dublin, Ireland; 4 Health Economics Unit, Centre for Health Policy, Melbourne School of Population and Global Health, The University of Melbourne, Melbourne, Australia

## Abstract

**Importance:**

Lifestyle interventions in pregnancy optimize gestational weight gain and improve pregnancy outcomes, with implementation recommended by the US Preventive Services Task Force. Yet, implementation research taking these efficacy trials into pragmatic translation remains limited.

**Objective:**

To evaluate success factors for implementing pregnancy lifestyle interventions into antenatal care settings in a meta-analysis, using the penetration, implementation, participation, and effectiveness (PIPE) impact metric.

**Data Sources:**

Data from a previous systematic review that searched across 9 databases, including MEDLINE, Embase, Cochrane Database of Systematic Reviews, Database of Abstracts of Reviews of Effects, Cochrane Central Register of Controlled Trials, and Health Technology Assessment Database, were obtained, in 2 stages, up to May 6, 2020.

**Study Selection:**

Randomized clinical trials reporting gestational weight gain in singleton pregnancies.

**Data Extraction and Synthesis:**

The association of penetration, implementation, and participation with effectiveness of antenatal lifestyle interventions in optimizing gestational weight gain was estimated using random-effects meta-analyses. The Cochrane risk of bias tool, version 1.0, was used to assess risk of bias.

**Main Outcomes and Measures:**

Penetration (reach), implementation (fidelity), participation, and effectiveness of randomized clinical trials of lifestyle interventions in pregnancy.

**Results:**

Ninety-nine studies with 34 546 participants were included. Only 14 studies reported penetration of target populations. Overall, 38 studies (38.4%) had moderate fidelity, 25 (25.2%) had high fidelity, and 36 (36.4%) had unclear fidelity. Participation was reported in 84 studies (84.8%). Lifestyle interventions were associated with reducing gestational weight gain by 1.15 kg (95% CI, –1.40 to –0.91 kg).

**Conclusions and Relevance:**

The findings of this systematic review and meta-analysis suggest that, despite the large body of evidence on efficacy of lifestyle interventions during pregnancy in optimizing gestational weight gain, little guidance is available to inform implementation of this evidence into practice. There is a need to better elucidate implementation outcomes in trial design alongside pragmatic implementation research to improve the health of women who are pregnant and the next generation.

## Key Points

**Question:**

Are antenatal lifestyle interventions for optimizing gestational weight gain in antenatal care settings implementable according to the penetration, implementation fidelity, participation, and effectiveness framework?

**Findings:**

In a meta-analysis of 99 randomized clinical trials in a systematic review assessing efficacy associated with antenatal lifestyle interventions, factors relating to broader implementation and scale-up were evaluated. Overall, penetration was reported in 14.1% of trials, fidelity was moderate or high in 63.3% of trials, and participation was moderate, with a mean of 49.0%.

**Meaning:**

The findings of this study suggest that evidence to support the capacity for implementation of antenatal lifestyle interventions to optimize gestational weight gain in antenatal care settings remains limited, with a need to improve rigor in reporting and conducting pragmatic implementation research to generate implementation learnings for broader benefit in antenatal care settings.

## Teaser

This meta-analysis examines the possibility for implementation of lifestyle interventions to optimize gestational weight gain in women who are pregnant.

## Introduction

Reproductive-aged women continue to have increasing obesity rates,^1^ with excess gestational weight gain (GWG) a key contributing factor.^2^ Universally endorsed guidelines by the National Academy of Medicine advise women who are pregnant on healthy GWG according to their preconception body mass index.^3^ Despite this advice, approximately 50% of women who are pregnant exceed recommended healthy GWG thresholds.^4^ Women with preconception obesity are more likely to exceed recommendations compared with their healthy weight counterparts.^3^ Excess GWG is associated with increased risk of adverse pregnancy outcomes, such as gestational diabetes, gestational hypertension, preeclampsia,^5^ macrosomia and large for gestational age birth, and cesarean delivery.^4^ Lifestyle interventions optimize healthy GWG and substantially improve pregnancy outcomes,^6^ with demonstrated cost-effectiveness.^7,8,9,10^ As such, there is a clear mandate to implement lifestyle interventions into practice to improve the health of mothers and babies for public health benefit, as recently emphasized by the US Preventive Services Task Force.^11^

Currently, strategies to ensure that evidence from randomized clinical trial interventions is successfully translated into practice with wide reach, broad impact, and sustained engagement remain unclear.^12^ Research translation is slow^13^ for a variety of reasons. One key barrier is that generating setting-specific implementation guidance from the limited contextual information within tightly internally controlled randomized clinical trials is challenging. However, impact metrics can be generated using standard trial reporting data to yield valuable implementation information to provide an indicative net public health benefit based on both intervention end user and clinician factors.^14^ Capturing impact metrics is conceptualized in the penetration (ie, the number and demographic characteristics of people reached in the target population), implementation (ie, the quality and degree of consistency to the key determinants of efficacy [ie, fidelity]), participation (ie, engagement and adherence in the intervention), and effectiveness (ie, pragmatic scalable impact in outcome measures) (PIPE) framework.^15^ The PIPE framework elements can be described both individually or combined to produce an overall metric (P × I × P × E), aiming to provide definitive insight to intervention performance and impact as well as areas for improvement and feasibility to inform implementation.^14^

With established efficacy of lifestyle interventions for both GWG and health outcomes, the imperative for implementation of evidence into practice exists, as emphasized in recent definitive systematic reviews^11,16^ in the field. Yet, to enable contextual adaptation for implementation and deliver population benefit, we must understand strategies that optimize penetration, retain implementation fidelity, and maximize participation alongside retained efficacy.^17,18^ For what we believe is the first time in this context, we aim to explore and understand the components of the PIPE framework within existing evidence to evaluate the potential of large-scale implementation of healthy lifestyle interventions in pregnancy into practice.

## Methods

### Search Strategy

This secondary analysis of a meta-analysis used the data sources from an original International Weight Management in Pregnancy systematic review and meta-analysis to 2017,^16,19,20^ with the protocol extended and the search updated in 2020.^16^ The primary systematic review and meta-analysis was prospectively registered in PROSPERO, and methods have been reported.^16^ Databases included MEDLINE, Embase, Cochrane Database of Systematic Reviews, Database of Abstracts of Reviews of Effects, Cochrane Central Register of Controlled Trials, and Health Technology Assessment Database, with searches completed May 6, 2020. Language restrictions were not applied to electronic searches. We followed the Preferred Reporting Items for Systematic Reviews and Meta-analyses (PRISMA) reporting guideline.^16^ Randomized clinical trials involving diet- and/or physical activity–based lifestyle interventions, with or without behavioral modification techniques, in women with singleton pregnancies were included. Control groups included women who were pregnant with no active lifestyle intervention and with routine antenatal care as defined by local health care practice. The primary outcome was mean GWG in kilograms. Studies were excluded if they included complicated pregnancies, were animal studies, focused on non–lifestyle-based components (ie, GWG monitoring only), and were published before 1990 when the National Academy of Medicine guidelines were first introduced.

### Study Selection

Two of us (M.B.K. and C.B.) independently evaluated each title and abstract for eligibility and reviewed full texts for inclusion criteria. Disagreements were resolved by another one of us (H.T.T.).^6,16,20^

### Quality and Bias Appraisal

Two of us (C.B. and an assistant) independently appraised methodologic quality^16^ using the Cochrane risk of bias tool, version 1.0,^21^ with a focus on 4 items: randomization, allocation concealment, blinding of outcome assessment, and incomplete outcome data. We ranked a study at high risk of bias if it scored high in at least 1 domain. A study was ranked as low risk of bias when all domains scored at low risk. All other studies were recorded as unclear. To assess publication bias, funnel plots were prepared, with GWG as the main outcome.

### PIPE and Original Systematic Review Data Extraction

One of us (M.B.K.) and an assistant extracted data on study characteristics including author, year of publication, country, main outcome of interest, target population, intervention timeframe, total target population number in a registry, number of participants reached /invited, number of participants enrolled, intended intervention and executed interventions, mean (SD) and significance of the GWG, and significance and frequency of gestational diabetes. Authors were contacted to supplement missing information. We categorized countries where the studies were performed according to the classification by the World Bank.^22^ Intervention type was independently identified by an experienced dietitian and an exercise physiologist; disagreements were resolved by one of us (H.J.T.). Classification into intervention types was described in detail previously.^16^ The PIPE Impact Metric was used to assess the program impact.^14^ Penetration was described as the proportion of the target population recruited with invitations to engage in interventions. Implementation in this framework captured factors aligned to intervention planning and fidelity. We evaluated this qualitatively based on 2 frameworks: standard curriculum comprising any descriptions of standard curriculum or intervention program manual to standardize interventions and limit discrepancies; and quality assurance across monitoring intervention delivery and specified training, checking consistency of delivery on session audio/video files or checklists documenting delivery.

Participation was based on the number of eligible participants who enrolled from those invited. Association was based on GWG, per the primary outcome of our systematic review. We further looked at the association of the intervention with significant reduction in the risk of gestational diabetes as a secondary outcome. The PIPE framework relies on numeric data to quantify results in the form of the PIPE Impact Metric, expressed in percentages.^14^

### Statistical Analysis

The PIPE Impact Metric elements were coded using the definitions presented in Table 1. Coefficients were calculated for penetration and participation. Intervention fidelity was ranked for implementation, by coding as high, medium, and unclear if relevant data were not available for ranking. For efficacy, mean difference with 95% CIs for GWG and the proportion of reduced risk with 95% CIs for gestational diabetes were estimated. For the outcome of GWG, a random effects meta-analysis applying the DerSimonian and Laird model was performed.^23^ To quantify statistical heterogeneity between studies, the *I*^2^ value was estimated for the efficacy in GWG; *I*^2^ greater than 50% implied substantial heterogeneity. Exploratory subgroup analyses were performed to explain sources of heterogeneity, including the main outcome of interest, country economy (ie, gross national income per capita less than $1045 considered low income; $1046-$4095, lower-middle income; $4096-$12 695, upper-middle income, and greater than $12 695, high income),^24^ target population body mass index category (overweight/obesity vs healthy), intervention type, penetration, implementation, and participation. Significance was defined as a 2-sided value of *P* < .05. All statistical analyses were performed using Stata, version 16 (StataCorp LLC).

**Table 1.** The PIPE Impact Metric Elements

Variable	Definition	Rate calculation	Coding
Penetration	Proportion of target population reached	No. of participants reached/invited ×100 Total No. of target population	Low: 0%-33% Moderate: 34%–66% High: 67%-100% NAC
Implementation	Fidelity: degree to which program was implemented according to plan		Moderate: standard curriculum or quality assurance High: standard curriculum and quality assurance Unclear: information not provided
Participation	Proportion of enrolled participants out of those reached	No. of participants enrolled ×100 No. of participants reached/invited	Low: 0%-33% Moderate: 34%-66% High: 67%-100% NAC
Effectiveness	Mean reduction in gestational weight gain (kilograms) Significant change in gestational weight gain		Significant: *P* < .05 for lower gestational weight gain Not reported (information not provided)

Abbreviation: NAC, not able to calculate.

## Results

Of a total of 7500 articles identified in the broader primary systematic review, 99 studies met inclusion criteria on GWG and formed the study data set (Figure). Study characteristics are presented in Table 2. Intervention types were structured diet in 13 studies,^25,27,31,44,59,60,80,86,106,114,118,120,123^ structured physical activity in 42 studies,^32,33,34,35,36,37,38,39,40,41,42,43,46,53,54,55,57,58,63,64,67,71,79,82,83,85,88,90,93,94,96,97,98,104,105,107,108,109,111,112,113,121^ diet with physical activity with at least 1 element structured in 16 studies,^29,48,49,51,52,62,65,73,74,84,95,99,101,110,117,119^ and mixed interventions that did not meet these criteria in 28 studies.^26,28,30,45,47,50,56,61,66,68,69,70,72,75,76,77,78,81,87,89,91,92,100,102,103,115,116,122^ Forty-one studies were conducted in Europe, 34 in the Americas, 13 in Oceania, and 11 in Asia. No studies were performed in low-income countries, 16 studies were performed in upper-middle income countries, and 83 studies were conducted in high-income countries. The smallest sample size was 12 participants^93^ and the largest sample size was 2261 participants.^87^ Women with overweight or obesity were recruited in 33.3% of the studies.

**Figure.**
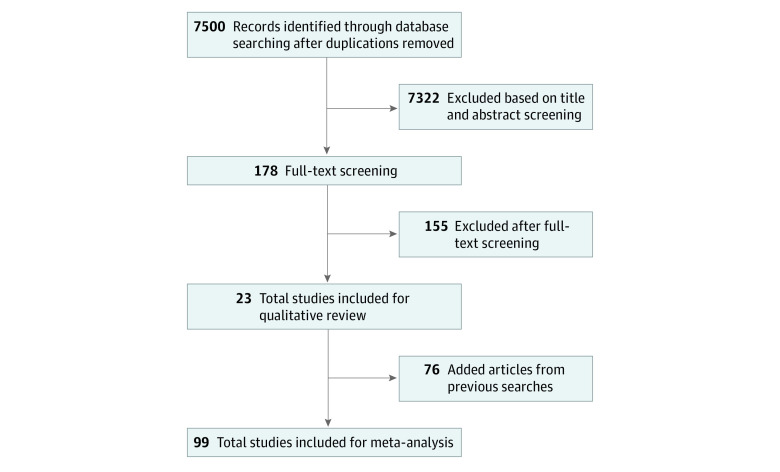
Flowchart of the Systematic Search Adapted from Teede et al.^16^

**Table 2.** PIPE of Randomized Clinical Lifestyle Trials in Women Who Are Pregnant Stratified by Intervention Type

Source	Intervention type	Sample size	Country	Risk of bias					Penetration	Implementation fidelity	Participation	Efficacy
				Randomization	Allocation concealment	Blinding of outcome assessment	Incomplete outcome data	Overall				
Al Wattar et al,^25^ 2019	Diet	1252	UK	Low	Low	Low	Low	Low	NAC	High	Low	Significant
Althuizen et al,^26^ 2013	Mixed	269	The Netherlands	Low	Low	Low	Low	Low	NAC	High	Moderate	Not significant
Anleu et al,^27^ 2019	Diet	1002	Chile	Low	Low	Low	Unclear	Unclear	NAC	Moderate	NAC	Not significant
Arthur et al,^28^ 2020	Mixed	396	Australia	Low	Low	High	Low	High	NAC	Unclear	Low	Not significant
Asbee et al,^29^ 2009	Diet with physical activity	100	US	Low	Low	Unclear	Unclear	Unclear	NAC	High	NAC	Significant
Aşcı et al,^30^ 2016	Mixed	90	Turkey	Low	Low	Unclear	Low	Unclear	High	Unclear	Moderate	Not significant
Assaf-Balut et al,^31^ 2017	Diet	874	Spain	Low	Low	High	Low	High	NAC	Unclear	Moderate	Significant
Bacchi et al,^32^ 2018	Physical activity	111	Argentina	Low	Low	High	High	High	NAC	Moderate	High	Not significant
Baciuk et al,^33^ 2008	Physical activity	70	Brazil	Low	Low	Low	Low	Low	NAC	High	High	Not Significant
Barakat et al,^34^ 2008	Physical activity	140	Spain	Unclear	Unclear	Low	Low	Unclear	NAC	Moderate	Low	Not significant
Barakat et al,^35^ 2011	Physical activity	67	Spain	Low	Unclear	Unclear	Low	Unclear	NAC	Moderate	Low	Significant
Barakat et al,^36^ 2012	Physical activity	83	Spain	Low	Unclear	Unclear	High	High	NAC	Moderate	Moderate	Significant
Barakat et al,^37^ 2012a	Physical activity	290	Spain	Low	Unclear	Unclear	Low	Unclear	NAC	Moderate	NAC	Not significant
Barakat et al,^38^ 2013	Physical activity	279	Spain	Low	Unclear	Unclear	High	High	NAC	Moderate	Moderate	Significant
Barakat et al,^39^ 2014	Physical activity	200	Spain	Low	Low	Unclear	Low	Unclear	NAC	Moderate	High	Significant
Barakat et al,^40^ 2016	Physical activity	765	Spain	Low	Low	Unclear	Low	Unclear	NAC	Moderate	High	Significant
Barakat et al,^41^ 2018	Physical activity	325	Spain	Low	Low	Unclear	Low	Unclear	NAC	Moderate	High	Significant
Barakat et al,^42^ 2019	Physical activity	520	Spain	Low	Low	Unclear	Low	Unclear	NAC	High	High	Significant
Bisson et al,^43^ 2015	Physical activity	45	Canada	Low	Low	Low	Low	Low	NAC	Moderate	NAC	Significant
Bechtel-Blackwell et al,^44^ 2002	Diet	46	US	High	Unclear	Unclear	High	High	NAC	Unclear	NAC	Not significant
Bogaerts et al,^45^ 2013	Mixed	197	Belgium	Low	Unclear	High	Low	High	NAC	Unclear	High	Significant
Brik et al,^46^ 2019	Physical activity	120	Spain	Low	Unclear	Unclear	Low	Unclear	NAC	High	Moderate	Not significant
Briley et al,^47^ 2002	Mixed	20	US	Unclear	Unclear	Unclear	High	High	NAC	Moderate	NAC	Not reported
Bruno et al,^48^ 2017	Diet with physical activity	131	Italy	Low	High	Low	Low	High	NAC	Unclear	High	Not significant
Buckingham-Schutt et al,^49^ 2019	Diet with physical activity	56	US	Unclear	Unclear	Unclear	Low	Unclear	NAC	Moderate	Moderate	Not significant
Cahill et al,^50^ 2018	Mixed	240	US	Low	Unclear	Low	Low	Unclear	NAC	High	Low	Significant
Chan et al,^51^ 2018	Diet with physical activity	229	China	Low	Low	Low	Low	Low	NAC	Moderate	Low	Not significant
Chao et al,^52^ 2017	Diet with physical activity	38	US	Low	Low	Unclear	Low	Unclear	NAC	High	Moderate	Not significant
Clapp et al,^53^ 2000	Physical activity	46	US	Low	Unclear	Unclear	Low	Unclear	NAC	Unclear	NAC	Not significant
Clark et al,^54^ 2019	Physical activity	42	US	Unclear	Low	Low	Low	Unclear	NAC	Moderate	High	Not significant
da Silva et al,^55^ 2017	Physical activity	594	Brazil	Unclear	Unclear	Unclear	Low	Unclear	NAC	Moderate	Low	Not significant
Daley et al,^56^ 2019	Mixed	616	UK	Low	Low	Unclear	Low	Unclear	High	High	Moderate	Not significant
Daly et al,^57^ 2017	Physical activity	76	Ireland	Low	Low	Low	Low	Low	NAC	Unclear	Low	Not significant
Dekker et al,^58^ 2015	Physical activity	35	Australia	Low	Unclear	Unclear	Unclear	Unclear	Moderate	Unclear	Moderate	Not significant
Deveer et al,^59^ 2013	Diet	100	Turkey	High	High	Unclear	Low	High	NAC	Moderate	NAC	Significant
Di Carlo et al,^60^ 2014	Diet	120	Italy	Unclear	Low	Low	Unclear	Unclear	NAC	Moderate	High	Significant
Dodd et al,^61^ 2014	Mixed	2199	Australia	Low	Low	Low	Low	Low	NAC	Moderate	Moderate	Not significant
Ferrara et al,^62^ 2020	Diet with physical activity	398	US	Low	Unclear	Low	Low	Unclear	NAC	High	Low	Significant
Garnæs et al,^63^ 2016	Physical activity	74	Norway	Low	Low	Low	Low	Low	NAC	Moderate	High	Not significant
Garshasbi et al,^64^ 2005	Physical activity	266	Iran	Unclear	Unclear	Unclear	Low	Unclear	Low	Unclear	High	Not significant
Gesell et al,^65^ 2015	Diet with physical activity	87	US	Low	Low	Unclear	High	High	NAC	High	Moderate	Not significant
Guelinckx et al,^66^ 2010	Mixed	195	Belgium	Low	Unclear	High	High	High	NAC	Unclear	High	Not significant
Haakstad et al,^67^ 2011	Physical activity	105	Norway	Low	Low	Low	Low	Low	NAC	Moderate	NAC	Not significant
Harrison et al,^68^ 2013	Mixed	238	Australia	Low	Low	Low	Low	Low	NAC	Unclear	Low	Significant
Hawkins et al,^69^ 2015	Mixed	68	US	Unclear	Unclear	Low	Low	Unclear	NAC	High	Low	Not significant
Herring et al,^70^ 2016	Mixed	56	US	Low	Low	Low	Unclear	Unclear	NAC	Moderate	Low	Not significant
Hopkins et al,^71^ 2010	Physical activity	84	New Zealand	Unclear	Unclear	Unclear	High	High	NAC	Unclear	Moderate	Not significant
Huang et al,^72^ 2011	Mixed	189	Taiwan	Low	Unclear	Low	High	High	Moderate	Unclear	Low	Significant
Hui et al,^73^ 2012	Diet with physical activity	183	Canada	Low	Unclear	Unclear	Low	Unclear	NAC	Moderate	High	Not significant
Hui et al,^74^ 2014	Diet with physical activity	113	Canada	Low	Unclear	Low	Low	Unclear	NAC	Moderate	High	Not significant
Jackson et al,^75^ 2011	Mixed	287	US	Low	Low	Unclear	Low	Unclear	NAC	High	Moderate	Not significant
Jeffries et al,^76^ 2009	Mixed	282	Australia	Low	Low	Low	Low	Low	NAC	Unclear	Moderate	Not significant
Jing et al,^77^ 2015	Mixed	221	China	Low	Unclear	Low	Low	Unclear	NAC	Unclear	NAC	Not significant
Kennelly et al,^78^ 2018	Mixed	535	Ireland	Low	Low	Unclear	Low	Unclear	NAC	High	Low	Significant
Khaledan et al,^79^ 2010	Physical activity	39	Iran	Low	Unclear	High	Low	High	NAC	Unclear	NAC	Not significant
Khoury et al,^80^ 2005	Diet	289	Norway	Low	Low	Low	Low	Low	NAC	Unclear	Low	Not significant
Kiani Asiabar et al,^81^ 2018	Mixed	150	Iran	Low	Unclear	Unclear	Low	Unclear	NAC	Unclear	High	Significant
Kihlstrand et al,^82^ 1999	Physical activity	241	Sweden	Low	Low	Unclear	Low	Unclear	Moderate	High	High	Not significant
Ko et al,^83^ 2014	Physical activity	1196	US	Low	Low	Unclear	Low	Unclear	NAC	Moderate	Low	Not significant
Koivusalo et al,^84^ 2016	Diet with physical activity	269	Finland	Low	Low	Unclear	Low	Unclear	NAC	High	Moderate	Significant
Kong et al,^85^ 2014	Physical activity	37	US	Low	Low	Unclear	Low	Unclear	NAC	Unclear	NAC	Not significant
Korpi-Hyövälti et al,^86^ 2012	Diet	54	Finland	Low	Low	High	Low	High	NAC	Unclear	Moderate	Not significant
Kunath et al,^87^ 2019	Mixed	2261	Germany	Unclear	Unclear	Unclear	Low	Unclear	NAC	High	High	Not significant
Marquez-Sterling et al,^88^ 2000	Physical activity	15	US	Unclear	Unclear	Unclear	High	High	NAC	High	NAC	Not significant
McCarthy et al,^89^ 2016	Mixed	371	Australia	Low	Low	Low	Low	Low	Low	Unclear	High	Not significant
Nascimento et al,^90^ 2011	Physical activity	80	Brazil	Low	Low	High	Low	High	NAC	Moderate	High	Not significant
Okesene-Gafa et al,^91^ 2019	Mixed	230	New Zealand	Low	Low	Low	Low	Low	NAC	High	Moderate	Not significant
Olson et al,^92^ 2018	Mixed	1689	US	Low	Unclear	Unclear	Low	Unclear	Moderate	Unclear	Low	Not significant
Ong et al,^93^ 2009	Physical activity	12	Australia	Low	Unclear	High	Low	High	NAC	Moderate	NAC	Not significant
Oostdam et al,^94^ 2012	Physical activity	105	The Netherlands	Low	Low	Low	High	High	Moderate	Moderate	Moderate	Not significant
Peaceman et al,^95^ 2017	Diet with physical activity	280	US	Low	Low	Low	Low	Low	NAC	High	Low	Significant
Pelaez et al,^96^ 2019	Physical activity	345	Spain	Low	Unclear	Unclear	Low	Unclear	NAC	Moderate	Moderate	Significant
Perales et al,^97^ 2015	Physical activity	167	Spain	Low	Unclear	Low	High	High	NAC	Moderate	High	Not significant
Perales et al,^98^ 2016	Physical activity	166	Spain	Low	Unclear	Low	High	High	NAC	Unclear	High	Not significant
Petrella et al,^99^ 2014	Diet with physical activity	61	Italy	Low	High	High	Low	High	NAC	Unclear	NAC	Not significant
Phelan et al,^100^ 2011	Mixed	393	US	Low	Low	Low	Low	Low	NAC	Unclear	Low	Not reported
Phelan et al,^101^ 2018	Diet with physical activity	256	US	Low	Unclear	Unclear	Low	Unclear	NAC	Unclear	Low	Significant
Polley et al,^102^ 2002	Mixed	110	US	Unclear	Unclear	Unclear	Low	Unclear	NAC	Moderate	High	Significant
Poston et al,^103^ 2015	Mixed	1554	UK	Unclear	Unclear	High	Unclear	High	High	Moderate	Low	Significant
Prevedel et al,^104^ 2003	Physical activity	39	Brazil	Low	Low	High	Unclear	High	High	Moderate	High	Not significant
Price et al,^105^ 2012	Physical activity	62	US	Low	Low	Unclear	High	High	NAC	Unclear	High	Not significant
Quinlivan et al,^106^ 2011	Diet	124	Australia	Low	Low	Low	Low	Low	NAC	Unclear	Low	Significant
Rodríguez-Blanque et al,^107^ 2020	Physical activity	162	Spain	Low	Unclear	High	Low	High	NAC	Moderate	Moderate	Significant
Ronnberg et al,^108^ 2015	Physical activity	374	Sweden	Low	Low	Low	Low	Low	High	Unclear	Low	Significant
Ruiz et al,^109^ 2013	Physical activity	927	Spain	Low	Unclear	Unclear	Low	Unclear	NAC	Moderate	Moderate	Significant
Sagedal et al,^110^ 2017	Diet with physical activity	600	Norway	Low	Low	Low	Unclear	Unclear	Moderate	High	Moderate	Significant
Santos et al,^111^ 2005	Physical activity	90	Brazil	Low	Unclear	Unclear	Unclear	Unclear	NAC	Unclear	Moderate	Not significant
Sedaghati et al,^112^ 2007	Physical activity	90	Iran	Unclear	Unclear	Unclear	High	High	Low	Moderate	High	Significant
Seneviratne et al,^113^ 2016	Physical activity	75	New Zealand	Low	Low	Unclear	Low	Unclear	NAC	Moderate	Moderate	Not significant
Sewell et al,^114^ 2017	Diet	28	UK	Low	Low	Unclear	Low	Unclear	NAC	High	Low	Not significant
Simmons et al,^115^ 2017	Mixed	436	UK	Low	Low	Low	Low	Low	NAC	High	Low	Significant
Smith et al,^116^ 2016	Mixed	45	US	Low	Low	Unclear	Low	Unclear	NAC	Moderate	Low	Not significant
Sun et al,^117^ 2016	Diet with physical activity	66	China	High	High	Unclear	High	High	NAC	Unclear	High	Significant
Thornton et al,^118^ 2009	Diet	232	US	Low	Unclear	Unclear	Low	Unclear	NAC	Unclear	Moderate	Significant
Vesco et al,^119^ 2014	Diet with physical activity	114	US	Low	Unclear	Low	Low	Unclear	NAC	High	Low	Significant
Walsh et al,^120^ 2012	Diet	759	Ireland	Low	Low	Unclear	Low	Unclear	NAC	Unclear	High	Significant
Wang et al,^121^ 2016	Physical activity	226	China	Low	Unclear	Unclear	Low	Unclear	NAC	Unclear	Moderate	Significant
Willcox et al,^122^ 2017	Mixed	91	Australia	Low	Low	Low	Low	Low	NAC	High	Low	Significant
Wolff et al,^123^ 2008	Diet	59	Denmark	Low	Low	High	High	High	NAC	Unclear	High	Significant

Abbreviations: NAC, not able to calculate; PIPE, penetration, implementation, participation, effectiveness.

The risk of bias assessment for the PIPE components in the subset of 99 studies on GWG is presented in Table 3. The risk of bias was low in 20 studies (20.2%), high in 30 studies (30.3%), and unclear in 49 studies (49.5%). Funnel plots of GWG suggested a possible publication bias for small studies that favored positive intervention group outcomes, confirmed by the Egger test.^16^

**Table 3.** Assessment of Lifestyle Intervention Types and Risk of Bias Over the PIPE Impact Metrics Elements of the 99 Trials Included in This Study

PIPE metrics	Intervention type				Risk of bias		
	Diet (n = 13)	Physical activity (n = 42)	Diet with physical activity (n = 16)	Mixed (n = 28)	Low (n = 20)	High (n = 30)	Unclear (n = 49)
Penetration							
NAC (n = 85)	13	35	15	22	18	25	42
Low (n = 3)	0	2	0	1	1	1	1
Moderate (n = 6)	0	3	1	2	0	2	4
High (n = 5)	0	2	0	3	1	2	2
Implementation							
Unclear (n = 32)	8	12	4	8	8	15	13
Moderate (n = 46)	3	25	4	14	5	13	20
High (n = 21)	2	5	8	6	7	2	16
Participation							
NAC (n = 15)	3	8	2	2	2	7	6
Low (n = 28)	4	6	5	13	11	3	14
Moderate (n = 27)	3	12	5	7	4	8	15
High (n = 29)	3	16	4	6	3	12	14
Effectiveness							
Not reported (n = 2)	0	0	0	2	1	1	0
Not significant (n = 57)	5	28	8	16	11	18	28
Significant (n = 40)	8	14	8	10	8	11	21

Abbreviations: NAC, not able to calculate; PIPE, penetration, implementation, participation, effectiveness.

Overall, 6 studies provided no information on penetration, implementation, and participation. They had unclear (n = 3)^53,77,85^ and high risk (n = 3)^44,79,99^ of bias. Seven studies provided all required information for the PIPE Impact Metric and they had either unclear (n = 3)^56,82,110^ or high (n = 4)^94,103,104,112^ risk of bias. Overall, these 7 studies had moderate penetration and participation and were associated with a reduction in GWG by 0.66 kg (95% CI, –1.17 to –0.16 kg). Of 13 studies with diet interventions, 9 studies (69.2%) provided information on only 1 component of PIPE, whereas 24 of 42 studies (57.1%) with physical activity, 10 of 16 (62.5%) with diet with physical activity, and 17 of 28 (60.7%) with mixed interventions provided information on 2 components.

### Penetration Rate

The number of invited participants was reported in 84 studies (84.8%); however, most studies (n = 85) did not specify the total size of the target population. Therefore, the penetration rate could not be calculated for most trials (85.9%). For studies in which the penetration rate could be determined (n = 14), penetration was moderate (50.0%) overall, ranging from 11.9% to 100%, with 3 studies classified as having low,^64,89,112^ 6 as moderate,^58,72,82,92,94,110^ and 5 as high^30,56,103,104,108^ penetration rates. Of these, 7 studies (50.0%) were physical activity interventions,^58,64,82,94,104,108,112^ 1 study (7.1%) was a diet with physical activity intervention,^110^ and 6 studies (42.9%) were mixed interventions^30,56,72,89,92,103^ (Table 3). Only 2 (14.3%) of the 14 studies had low risk of bias,^89,108^ with 1 classified as having a high penetration rate^108^ (Table 3). Most of the studies with insufficient data for penetration calculation had unclear and high risk of bias (78.8%).

### Implementation

Of 99 studies, 67 studies (67.7%) reported some form of fidelity check. Overall, 38 studies (38.4%) studies had moderate fidelity, 25 (25.2%) had high fidelity, and 36 (36.4%) had unclear fidelity. At least 1 fidelity component (moderate to high fidelity) was reported by 5 studies (7.9%) with diet interventions, 30 (47.6%) with physical activity interventions, 12 (19.1%) with diet with physical activity interventions, and 16 (25.4%) with mixed interventions (Table 3). The proportion of studies with both standard curriculum and quality assurance measures was the highest among diet with physical activity interventions (8 [50.0%]) and lowest for physical activity interventions (5 [11.9%]). However, the proportion of studies following a standard curriculum was the highest in physical activity interventions (29 [57.1%]). The majority of studies with diet (8 [61.5%]) and mixed (12 [42.9%]) interventions had neither a standard curriculum nor quality assurance measures. Most studies with high fidelity (16 [64.0%]) had unclear risk of bias; most studies with unclear fidelity (28 [77.8%]) had an unclear and high risk of bias (Table 3).

### Participation

Participation was well reported, with Consolidated Standards of Reporting Trials data completed for most studies. Of 99 studies, we were able to retrieve participation data from 84 (84.9%) studies. Participation rate was moderate (49.0%), ranging from 3% to 100%. Overall, 29 studies (29.3%) had high participation, 27 (27.3%) had moderate participation, and 28 (28.3%) had low participation rates. The participation rate was 45% for diet interventions (calculable for 76.9% studies), 59% for physical activity interventions (calculable for 81.0% studies), 44% for studies with diet with physical activity interventions (calculable for 87.5% studies), and 39% for studies with mixed interventions (calculable for 92.9% studies) (Table 3). Most studies with high participation rate (26 [89.7%]) had unclear and high risk of bias; studies with low participation rate (11 [39.3%]) had the highest rate of low risk of bias (Table 3).

### Effectiveness

As previously reported,^16^ lifestyle intervention was associated with a reduction in GWG of 1.15 kg (95% CI, –1.40 to –0.91 kg) compared with controls, with all intervention types shown to be efficacious. Diet interventions were associated with a reduction in GWG of 2.63 kg (95% CI, –3.87 to –1.40 kg); physical activity by 1.04 kg (95% CI, –1.33 to –0.74 kg); diet and physical activity interventions by 1.35 kg (95% CI, –1.95 to – 0.75 kg) and mixed interventions by 0.74 kg (95% CI, –1.06 to –0.43 kg) (Table 3). Subgroup analysis by risk of bias showed studies with high risk of bias were associated with a marginally higher efficacy (–1.23 kg; 95% CI, –1.75 to –0.70 kg). Overall, 40 of 99 individual studies (40.4%) were associated with improved GWG and, of these, 8 (20.0%) had low and 21 (52.5%) had unclear risk of bias. Substantial heterogeneity was found between studies (*I*^2^ = 85.3%) as well as by intervention types (*I*^2^>50%).^16^

### Association of Lifestyle Interventions With GWG Optimization

On evaluation, heterogeneity in efficacy could not be explained by main outcome of interest, country economy, target population’s body mass index category, intervention type, implementation (fidelity) measures, and participation rate (*I*^2^>50%). With limited studies reporting the required information for penetration assessment, we were underpowered for evaluation of subgroups of low, moderate, and high participation.

Studies with high fidelity were associated with reducing GWG by 0.94 kg (95% CI, –1.31 to –0.56 kg), moderate fidelity by 1.18 kg (95% CI, –1.57 to –0.80 kg), and unclear fidelity by 1.31 kg (95% CI, –1.81 to –0.81 kg) with substantial heterogeneity present (*I*^2^≥59.5%). Of the 56 articles that reported the use of a standard curriculum, 24 studies (42.9%) showed significant efficacy in GWG (–1.11 kg; 95% CI, –1.40 to –0.81 kg). Of 32 articles that reported use of quality assurance, 15 studies (46.9%) showed significant reduction in GWG (–0.88 kg; 95% CI, –1.23 to –0.53 kg).

Studies with a high participation rate were associated with reducing GWG by 1.15 kg (95% CI, –1.61 to –0.69 kg), moderate participation rate by 1.04 kg (95% CI, –1.59 to –0.49 kg), low participation rate by 1.21 kg (95% CI, –1.64 to –0.77 kg), and insufficient data for participation by 1.31 kg (95% CI, –2.09 to –0.53 kg), with substantial heterogeneity in subgroup analysis (*I*^2^≥64.4%). As reported, studies with a low risk of bias were associated with reducing GWG by 1.13 kg (95% CI, –1.63 to –0.63 kg), high risk of bias by 1.23 kg (95% CI, –1.75 to –0.70 kg), and unclear risk of bias by 1.13 kg (95% CI, –1.48 to –0.78 kg) with substantial heterogeneity in subgroup analysis (*I*^2^≥73.2%).^16^

## Discussion

To our knowledge, this is the first PIPE framework evaluation of lifestyle interventions in pregnancy to optimize GWG and maternal outcomes. Across 99 studies involving 34 546 pregnancies, only 14.1% of included randomized clinical trials reported penetration into the target population. Approximately two-thirds of studies reported at least 1 form of fidelity measure and were rated as having moderate to high fidelity. Most studies provided sufficient data to calculate participation rate and most had moderate to high participation, particularly physical activity interventions. Compared with the other approaches, diet interventions were associated with greater reduction of GWG, and physical activity interventions were associated with greater reduction in gestational diabetes. Only 20.2% of studies were of low risk of bias for the outcome of GWG. On evaluation, sources of heterogeneity between studies could not be explained by exploratory variables including outcome of interest, country economy, target population body mass index category, intervention type, degree of implementation fidelity measure, and participation rate.

The delay between efficacy-based controlled trial research and its translation to implementation to the benefit of broader populations and end users is an established research concept. There are currently more than 117 diverse randomized clinical trials of antenatal lifestyle interventions spanning 5 continents and 3 decades of research. When evaluated individually, efficacy between trials varies, emphasizing that a universal approach does not exist, rather, individual trials contribute to a cyclic knowledge and learning bank^12^ and, when pooled, a more definitive understanding may be determined. A recent pooled meta-analysis^16^ described an association between antenatal lifestyle interventions and optimizing total GWG and associated maternal outcomes independent of concordance with National Academy of Medicine–recommended thresholds, supporting the adoption of healthy lifestyle interventions in pregnancy.^16^ Yet herein, in exploring factors related to the capacity for implementation of interventions, we found poor reporting for penetration (reach) within a given population, high intervention fidelity in only 21% of interventions, and high participation in 29% of interventions. In addition, an unclear or high risk of bias was observed across most studies. For these reasons, the PIPE metric for antenatal lifestyle interventions could not be determined and sources of heterogeneity could not be elucidated. This is a major barrier to the recent US Preventive Services Task Force recommendation advocating implementation and highlights the need for improved study design and reporting. With limited implementation knowledge generated, translation of trials into settings remains delayed.

The need for better trial and intervention reporting is widely advocated.^124^ A recent review^12^ scoping factors related to reporting and the capacity to implement interventions emphasizes that, with rigorous reporting, individual trials can contribute to activities and learnings related to implementation, irrespective of whether the trials are designed for implementation. The review identified concepts considered important to trial reporting, including provision of sufficient information to assess applicability, replicate the intervention, and assess the risk of bias within the trial.^12^ Herein, we found that a third of the studies did not report measures for fidelity, such as standard curriculum and quality assurance measures, and of those reporting a minimum of 1 fidelity measure, most were deemed unclear or at high risk of bias. This result may increase the risk of reporting and/or performance bias, which may in part explain higher reported efficacy in trials with increased risk of bias. Although not currently a requirement for intervention studies, adoption of frameworks and/or checklists promoting better reporting will theoretically address such risks of bias while also improving access to possible implementation of information. Such checklists include the Standard Protocol Items: Recommendations for Interventional Trials statement and the Template for Intervention Description and Replication (TIDieR) guideline and checklist. The Template for Intervention Description and Replication was developed to better support items related to study replicability within the Consolidated Standards of Reporting Trials statement and provides items related to why (ie, informing applicability), what (ie, materials and procedure), who (ie, facilitator or provider), how (ie, delivery mode and format [group or individual]), where (ie, setting), when (ie, timeframe), and how much (ie, intensity or frequency), tailoring, modifications, and fidelity measures including a review of planned and actual intervention activities.^124,125^ Incorporating rigorous reporting and adoption of such frameworks is essential to better understand strategies that optimize penetration, implementation, participation, and efficacy. With efficacy established, yet limited implementation knowledge available, pragmatic implementation feasibility trials to address these elements are required to deliver return on research investment^126,127^in antenatal lifestyle interventions and provide implementation knowledge and assessment of generalizability to antenatal care settings.^128^

### Strengths and Limitations

The strengths of the present study include building on a robust systematic review and meta-analysis and intervention categorization, evaluating the association of lifestyle interventions with GWG, and applying the established PIPE framework. Eligibility criteria included usual care as the comparator with the intervention group, which increases the generalizability of the results to antenatal care settings.^17^

Limitations of the study include inconclusive understanding of the capacity for implementation of the lifestyle interventions in pregnancy, owing to lack of inclusion of the PIPE Impact Metric within studies. This may, in part, be owing to a lack of a standard curriculum for measuring all metrics contained within PIPE, including fidelity of antenatal lifestyle interventions, compared with other fields on which the PIPE framework is based, such as the US Diabetes Prevention Program. In addition, we did not evaluate approaches used to inform penetration and participation, including recruitment methods within trials, that may have potentially explained variability in the respective impact metrics reported. Substantial heterogeneity in effectiveness could not be tested by penetration rate and could not be explained by fidelity and participation based on reported data. Furthermore, most studies had high or unclear risk of bias. A major publication bias was found against effectiveness in small studies, which might have increased the effect size; however, similar findings have been reported for GWG when analyzing studies with low risk of bias.^16^

## Conclusions

Although antenatal lifestyle interventions are associated with optimizing GWG and improving associated maternal and neonatal outcomes, implementation knowledge across penetration, implementation fidelity, and participation is limited, curtailing translation and scale-up for population-level impact. Capturing implementation learnings across trial design, conduct, and reporting needs greater consideration alongside pragmatic implementation research to improve the health of women who are pregnant and the next generation.
